# Impact of Renal Sympathetic Denervation on Left Ventricular Structure and Function at 1-Year Follow-Up

**DOI:** 10.1371/journal.pone.0149855

**Published:** 2016-03-02

**Authors:** Manuel de Sousa Almeida, Pedro de Araújo Gonçalves, Patricia Branco, João Mesquita, Maria Salomé Carvalho, Helder Dores, Henrique Silva Sousa, Augusta Gaspar, Eduarda Horta, Ana Aleixo, Nuno Neuparth, Miguel Mendes, Maria João Andrade

**Affiliations:** 1 Hospital de Santa Cruz, Lisbon, Portugal; 2 Hospital da Luz, Lisbon, Portugal; 3 CEDOC- Nova Medical School, Lisbon, Portugal; University Medical Center Utrecht, NETHERLANDS

## Abstract

**Background:**

Catheter-based sympathetic renal denervation (RDN) is a recent therapeutic option for patients with resistant hypertension. However, the impact of RDN in left ventricular (LV) mass and function is not completely established. Our aim was to evaluate the effects of RDN on LV structure and function (systolic and diastolic) in patients with resistant hypertension (HTN).

**Methods and Results:**

From a single centre prospective registry including 65 consecutive patients with resistant HTN submitted to RDN between July-2011 and April-2015, 31 patients with baseline and 1-year follow-up echocardiogram were included in this analysis. Mean age was 65±7 years, 48% were males, 71% had type 2 diabetes. Most had hypertension lasting for more than 10 years (90%), and were being treated with a median number of 6 anti-hypertensive drugs, including 74% on spironolactone. At 1-year, there was a significant decrease both on office SBP (176±24 to 149±13mmHg, p<0.001) and DBP (90±14 to 79±11mmHg, p<0.001), and also in 24h ABPM SBP (150±20 to 132±14mmhg, p<0.001) and DBP (83±10 to 74±9mmHg, p<0.001). There was also a significant decrease in LV mass from 152±32 to 136±34g/m^2^ (p<0.001), an increase in LV end diastolic volume (93±18 to 111±27 mL, p = 0.004), an increase in LV ejection fraction (65±9 to 68±9%, p = 0.001) and mitral valve E deceleration time (225±49 to 247±51ms, p = 0.015) at 1-year follow up. There were no significant changes in left atrium volume index or in the distribution of patients among the different left ventricle geometric patterns and diastolic function subgroups.

**Conclusions:**

In this single centre registry of patients with resistant hypertension, renal denervation was associated with significant reduction in both office and ABPM blood pressure and a significant decrease in left ventricle mass evaluated by transthoracic echocardiogram at 1 year follow-up.

## Introduction

Long-standing hypertension (HTN) results in cardiac remodelling including myocardial hypertrophy, diastolic dysfunction and left atrial (LA) enlargement leading to atrial and ventricular arrhythmias, heart failure and ultimately to myocardial infarction and stroke, which are the leading causes of death and morbidity in developed countries [1].

The link between chronic sympathetic hyperactivity and drug-resistant HTN is well known for several years, and is the rationale behind the development of catheter-based sympathetic renal denervation (RDN). This treatment approach for drug resistant HTN had very promising results in early non-blinded studies [2,3]. Recently, the lack of positive results on a randomized sham-controlled trial raised doubts on the efficacy and patient selection for this procedure, reinforcing the need for further research in this field [4]. Sympathetic drive is also implicated in the development of left ventricular hypertrophy (LVH) [5,6], but little is known about the impact of RDN in left ventricular performance. The aim of the present study was to evaluate the effects of RDN on LV structure and function (systolic and diastolic) in patients with resistant HTN.

## Methods

### Study design and population

**Fig 1:**
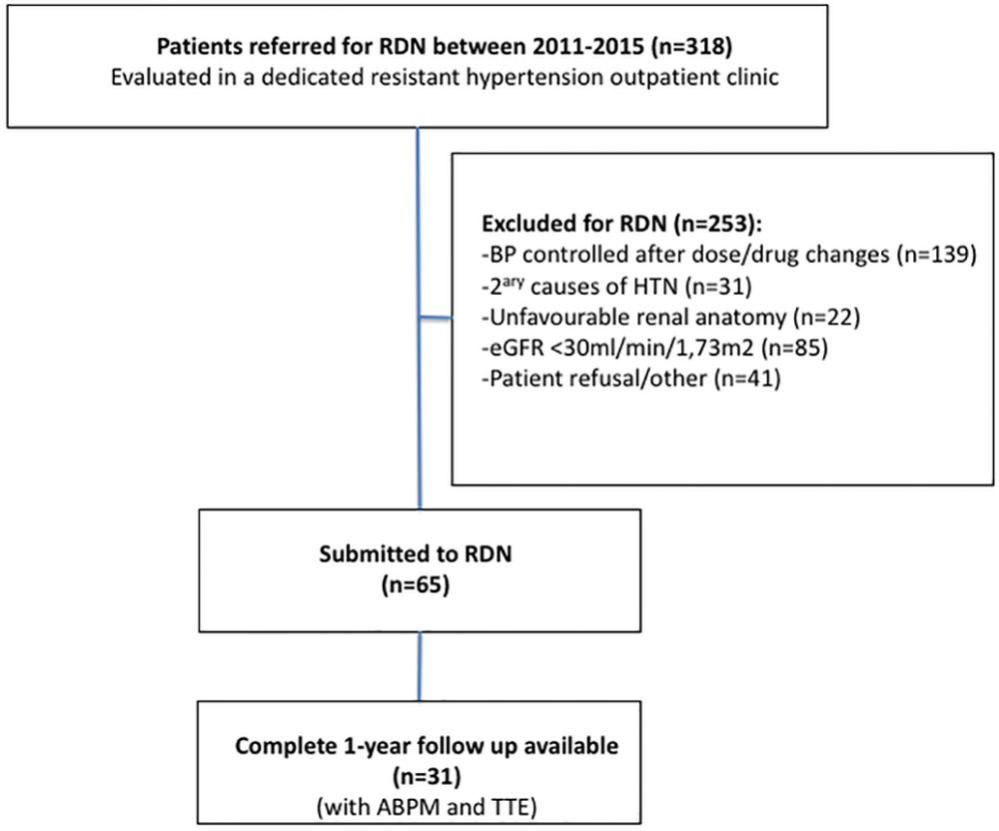
Flowchart with patient selection. From the total number of patients evaluated in a dedicated outpatient hypertension clinic (n = 318), 65 patients were submitted to renal denervation, after the exclusion of 253 due to several reasons listed. From these 65 patients, it was possible to obtain complete 1 year follow up with ambulatory blood pressure measurement and transthoracic echocardiogram. RDN—renal denervation; HTN—hypertension; eGFR—estimated glomerular filtration rate; ABPM –24 hours ambulatory blood pressure measurement; TTE-transthoracic echocardiogram.

From a single centre prospective registry including 65 consecutive patients with resistant HTN submitted to RDN between July-2011 and April-2015, 31 patients with baseline and 1-year follow-up 24h ABPM and transthoracic echocardiogram were included in this analysis. As per protocol, all patients underwent a comprehensive transthoracic echocardiogram (TTE) at baseline and at 1-year after RDN. The inclusion, exclusion criteria and clinical feature regarding this registry were previously reported [7]. The research was approved by the Ethics committee of Hospital de Santa Cruz and Nova Medical School, Lisbon, Portugal. Written informed consent was collected for all the patients. Study design is summarized in Fig 1.

In summary, the patients selected had to be older than 18 years, with an office systolic blood pressure (SBP) above 160mHg while receiving a stable antihypertensive regimen involving at least three drugs (including a diuretic). Before RDN, during pre-scheduled visits at the outpatient clinic for a period not less than 6 weeks, secondary causes for HTN were excluded, compliance to medical treatment was assured and drug therapy was adjusted until maximal tolerated regimens. Only then, if target BP values were not obtained, patients were considered for RDN. Anatomical criteria were adopted from Symplicity trials.[2,8] Demographic variables, clinical characteristics, anthropometric data, laboratory values, drug treatment and procedure details were recorded and stored in a dedicated database. Creatinine clearance was calculated using MDRD formula.[9]

### Blood pressure measurement and definition of responders

Office BP readings were taken in a seated position with an oscillometric semiautomatic sphygmomanometer Omron HEM-907 monitor (Omron Healthcare, USA) after 5 min of rest according to the European Guidelines for the management of arterial hypertension [10] At baseline, BP was measured in both arms and the arm with the higher BP was used for all subsequent readings. Averages of the triplicate measures were calculated and used for analysis.

Twenty-four hours ambulatory blood pressure measurements (ABPM) were taken with an ABM monitor (Spacelabs Healthcare, USA), according to the current European Society of Hypertension guidelines[10].

Blood pressure responders to RDN treatment were defined as those who had a reduction in office SBP of ≥10 mmHg at one year follow-up or a reduction of 2mmHg in ABPM 24 hours SBP according to Symplicity HTN3 trial design[11].

### Renal denervation procedure

We have previously reported the details of the RDN procedure in our center [12]. Briefly, all procedures were performed under mild anaesthesia for sedation and pain control (propofol and remifentanil in weight-adjusted doses). Anticoagulation with unfractionated heparin was used in order to obtain an activated clotting time between 250–300 seconds. After gaining femoral artery access in all cases except one (where the radial artery was used), abdominal aortography and selective renal artery angiograms were performed to confirm anatomic eligibility. At the end, in cases with femoral access, the site was closed using a sealing device (Angio-Seal^®^ -St. Jude Medical, USA).

RDN was performed using the Symplicity^®^ (n = 25), the EnligHTN^®^ (n = 4), OneShot^®^ (n = 2) catheter using the standard technique, as previously reported [7,12,13].

### Transthoracic echocardiography

Comprehensive two-dimensional and Doppler transthoracic echocardiographic studies were performed at baseline and at 1-year follow-up in all patients, using VIVID 7 ultrasound system (General Electric Heathcare). All echocardiographic recordings were stored in digital format on a dedicated workstation for off-line subsequent analysis. The exams were performed by one of two experienced operators (EH and MJA), while analysed and interpreted by the non-performer operator, both blinded to patients’ clinical, BP status and sequence of images.

Left ventricular size was evaluated by both linear (using M-mode 2D guided diameters obtained perpendicular to the LV long axis) and volumetric (using the biplane method of disks summation from tracings of the blood-tissue interface in the apical four- and two-chamber views), according to accepted recommendations from the American Society of Echocardiography and the European Association of Cardiovascular Imaging [14]. LV ejection fraction was calculated using the following formula: EF = (EDV − ESV)/EDV, with LV volume estimates obtained by the biplane method of disks.

Assessment of LV mass (LVM) was performed by the linear method using the cube formula (LV mass = 0.8 · 1.04 · [(IVS + LVID + PWT)3 −LVID3]+0.6g), with 2D guided M-mode measurements obtained at end-diastole from the parasternal approach perpendicular to the LV long axis measured at the level of the mitral valve leaflet tips. LV hypertrophy was considered present when LV mass exceeded 115 g/m^2^ for men and 95 g/m^2^ for women.

We also calculated the relative wall thickness (RWT) measured as twice the posterior wall thickness divided by left ventricular end-diastolic diameter, and determined the LV anatomical pattern in each participant. Normal LVM and RWT were defined as normal LV anatomy, normal LVM and RWT >0.42 as concentric LV remodeling, increased LVM and RWT >0.42 as concentric LVH and increased LVM in the presence of RWT <0.42 as eccentric LVH [15]. Left atrial (LA) volume measurement was done using the disk summation algorithm similar to that used to measure LV volume, when the LA chamber was at its greatest dimension (end of LV systole).

LV diastolic function was assessed by pulsed-wave Doppler examination of mitral inflow and Doppler tissue imaging of the mitral annulus. Peak velocities of early (E) and late (A) trans-mitral flow and deceleration time (DT) were determined, and the ratio E/A was calculated. Doppler tissue imaging with pulsed-wave Doppler at the level of septal and lateral mitral annulus was used to measure e’ velocities. The average of septal and lateral mitral annulus e’ peak velocities, were used to calculate the E/e’ ratio. The Valsalva maneuver was performed to distinguish normal from pseudo-normal patterns. Spectral recordings were obtained at a sweep speed of 100 mm/s at end-expiration, and each measurement was averaged over multiple cardiac cycles to account for inter-beat variability.

Grade 1 diastolic dysfunction (impaired relaxation) was defined by the presence of an E/A ratio <0.8, a deceleration time >200 ms and E/e′ ratio <8 in the presence of an enlarged left atrium. Moderate (pseudo-normal, grade 2) diastolic dysfunction was defined as a mitral E/A ratio >0.8 and <1.5 that decreases by 50% during the Valsalva maneuver, E/e’ ratio 9 to 12 and e’<8 cm/s. Finally, severe (grade 3) diastolic dysfunction corresponds to restrictive LV filling defined by E/A ratio >2, DT <160 ms, and average E/e’>13. All subjects with impaired LV relaxation, pseudo-normal or restrictive filling patterns were defined as having LVDD [16].

### Statistical analyses

Continuous variables are reported as mean ± standard deviation. Normality was tested with the Kolmogorov-Smirnov test and/or Q-Q Plot visual assessment. Normally distributed variables were compared between baseline and one year follow-up using a paired Student t test or a Wilcoxon matched-pairs test if not normally distributed. Discrete variables are expressed as frequencies and percentages (in brackets). Statistical comparisons of baseline characteristics and at follow-up were performed using the chi-square test or Fisher’s exact test, when appropriate, for categorical variables and the paired *t*-Student’s test or the Saterwate test for continuous variables. A two-tailed p value <0.05 is considered as statistically significant. Linear regression analyses were used to calculate the correlation between the change in blood pressure and the change in echocardiographic parameters. SPSS, Statistical Package for the Social Sciences^®^, V.21.0 (IBM SPSS Inc, Chicago, IL) software was used for data processing and statistical analysis.

## Results

### Patient characteristics

From the total number of patients evaluated in a dedicated outpatient hypertension clinic (n = 318), 65 patients were submitted to renal denervation, after the exclusion of 253 due to several reasons (listed in Fig 1). From these 65, it was possible to obtain complete 1 year follow up with ambulatory blood pressure measurement and transthoracic echocardiogram in 31 patients that were the final population included in this analysis. Mean age was 65±7 years, 48% were males (n = 15), and all were caucasians. Concerning traditional cardiovascular risk factors, obesity was present in 68% of the patients (mean body mass index 32±6 Kg/m^2^), type 2 diabetes in 71%, dyslipidaemia in 68% and active smoking in one patient (3.2%). Coronary artery disease was present in 10 patients (32%) and any vascular disease in 11 (36%). Estimated mean glomerular filtration rate was 76±25mL/min/1.73m^2^, with five patients having chronic kidney disease, defined as eGFR <60 ml/min/1.73 m^2^. Baseline characteristics are shown in Table 1.

**Table 1:** Patient’s baseline and RDN procedure characteristics.

**Demographic and clinical variables**	
Age (years)	65±7
Male (%)	15 (48.4)
Caucasians (%)	31 (100)
Weight (kg)	86±16
Height (m)	1.65±0.1
BMI (kg/m^2^)	31.8±5.5
Obesity (%)	21 (67.7)
Atrial fibrillation (%)	1 (3.2)
Previous stroke (%)	2 (6.5)
Type 2 Diabetes (%)	22 (71)
Dyslipidaemia (%)	21 (67.7)
Smoking (%)	1 (3.2)
Sleep apnea (%)	5 (19.1)
eGFR (ml/min/1,73m^2^)	76.4±24.7
CKD* (%)	5 (16.1)
Hypertension > 10 years (%)	28 (90.3)
Coronary artery disease (%)	10 (32.3)
Any vascular disease (%)	11 (35.5)
**RDN Procedure**	
Mean number of applications right renal artery	5.1±1.3
Mean number of applications left renal artery	5.7±1.1
Mean number of applications per patient	10.8±2.3

eGFR, estimated glomerular filtration rate;

CKD, *Chronic kidney disease(eGFR <60 ml/min/1,73m^2^)

The majority of patients (90%) had hypertension lasting for more than 10 years, treated with a median of 5.8 anti-hypertensive agents from a median of 5.5 different pharmacological classes. Almost all patients were treated with calcium antagonists, 96.8% (n = 30), 87% with diuretics, 74% with spironolactone, 61% with ACE inhibitors, 61% with ARB inhibitors, 84% with beta-blockers and 71% with a sympatholytic drug. (Table 2)

**Table 2:** Antihypertensive medication.

	Baseline	One year	p
Mean number of antihypertensive drugs	5.8±1.1	5.0±1.2	0.002
Mean number of classes	5.5±0.9	4.9±1.1	0.015
ACE inhibitors	19 (61.3)	17(54.8)	0.688
ARBs (%)	19 (61.3)	18 (58.1)	1.0
Beta-blockers (%)	26 (83.9)	27 (87.1)	1.0
Calcium channel blockers (%)	30 (96.8)	21 (67.7)	0.012
Diuretics (%)	27 (87.1)	24 (77.4)	0.727
Spironolactone (%)	23 (74.2)	26 (83.9)	0.453
Sympatholytic (%)	22 (71)	19 (61.3)	0.508
Aliskirene	4 (12.9)	0	0.046

ACE, Angiotensin converting enzyme; ARB, Angiotensin receptor blockers

### Blood pressure control by RDN

At baseline, mean office SBP and diastolic blood pressure (DBP) were 176±24 mmHg and 90±14 mmHg, respectively, and mean heart rate was 73±11 bpm. The 24-hour ABPM showed the following average values: SBP 150±20 mmHg, DBP 83±10 mmHg, pulse pressure 67±18mmHg (Table 3).

**Table 3:** RDN results on blood pressure and heart rate.

	Baseline	One-year	P
Office systolic BP (mmHg)	176±24	149±13	< .001
Office diastolic BP (mmHg)	90±14	79±11	< .001
Heart rate (bpm)	73±11	70±11	.261
ABPM systolic BP (mmHg)	150±20	132±14	< .001
ABPM diastolic BP (mmHg)	83±10	74±9	< .001
ABPM pulse pressure (mmHg)	67±18	58±13	.001
ABPM mean pressure (mmHg)	105±9	95,3±8,4	< .001
ABPM heart rate (bpm)	67.6±9.1	65.5±9.5	.090
ABPM SBP responders* (%)	-	26 (83.9)	-
Office SBP responders** (%)	-	22 (71)	-

BP, blood pressure; bpm, beats per minute; ABPM, 24 hours ambulatory blood pressure measurement;

* ABPM SBP responders: a decrease of 2mmHg between baseline ABPM SBP and at one year;

**Office SBP responders: a decrease of 10mmHg between baseline office SBP and at one year.

**Fig 2:**
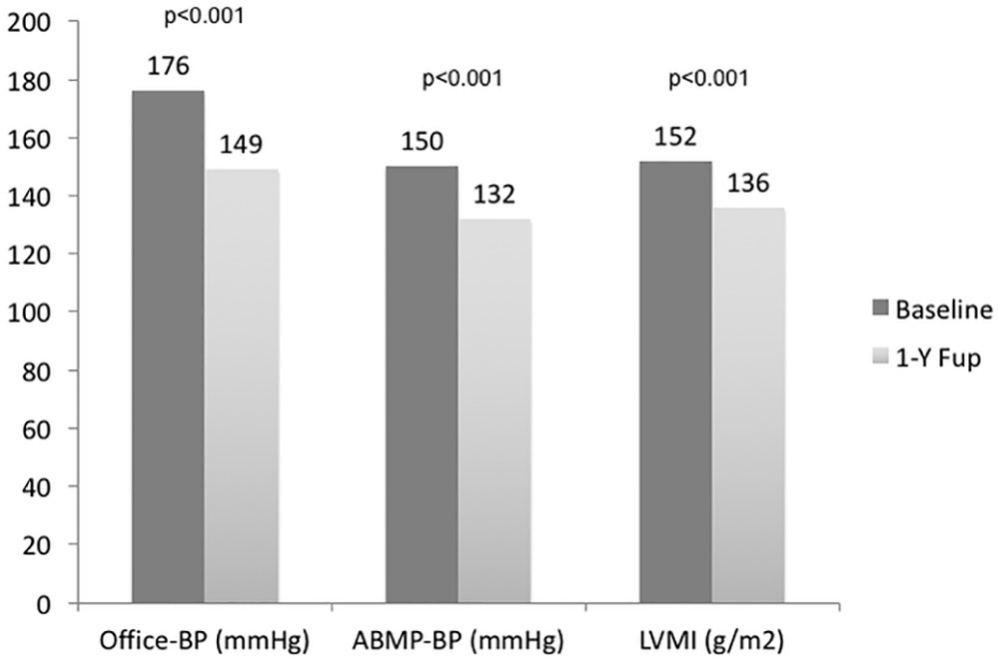
Results at 1 year after renal denervation (blood pressure and left ventricle mass index). Results in systolic blood pressure (both office and ABPM) and LVMI in TTE at 1-year follow-up are shown, with significant reductions in both parameters. BP- blood pressure; ABPM –24 hours ambulatory blood pressure measurement; LVMI—left ventricle mass index; TTE-transthoracic echocardiogram.

Overall, at one-year follow-up, there was a significant reduction in both office SBP (176±24 to 149±13mmHg, p<0.001) and DBP (90±14 to 79±11mmHg, p<0.001). On 24-hour ABPM, there was a significant drop on: SBP (150±20 to 132±14 mmHg, p<0.001, mean decrease of 18 mmHg), on DBP (83±10 to 74±9 mmHg, average decrease of 9 mmHg, p<0.01) and on pulse pressure from 67±18 to 58±13 mmHg, p = 0.001, a mean decrease of 5 mmHg (Fig 2).

This was found in spite of the significant reduction in the number of both antihypertensive drugs and classes in use at 1-year: 5.8±1.1 to 5.0±1.2 (p = 0.002) and 5.5±0.9 to 4.9±1.1 (p = 0.015) respectively.

At 1-year follow-up, 22 of patients (71%) were considered office SBP responders and 26 (84%) ABPM SBP responders based on a drop of more than 10mmHg on office SBP and 2mmHg on 24 hours ABPM SBP.

### Echocardiographic parameters

**Fig 3:**
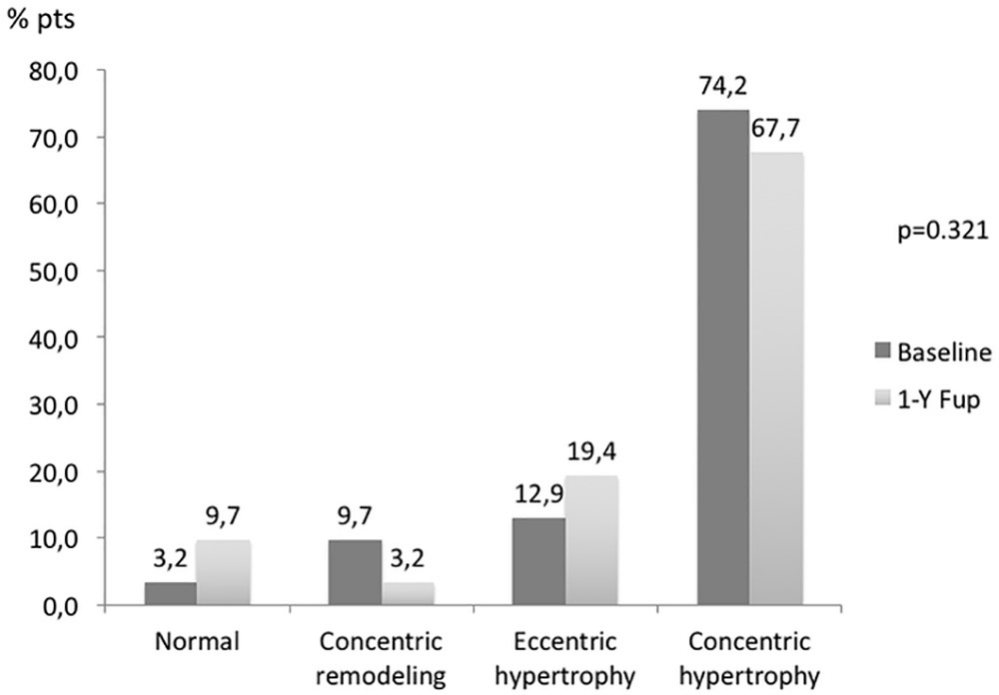
Comparison of different LV geometric patterns at baseline and 1 year after renal denervation. The percentage of patients in each LV geometric pattern class is depicted. Concentric remodelling was defined as relative wall thickness (RWT) of >0.42 with normal LV mass and normal geometry was defined as a RWT of ≤0.42 with normal LV mass.

Transthoracic echocardiography at baseline revealed LV hypertrophy in 27 patients (87%), with a mean LV mass of 152±32 g/m^2^. Distribution among different geometric patterns is shown in Fig 3. The large majority had concentric hypertrophy (74%), with only 3% presenting a normal pattern. All patients had a preserved EF (>55% by Simpson’s biplane method), with a mean LVEF of 65±9%. Mean LA volume was 33±8mL/m^2^, and 48.4% had ≥ 34ml/ m2.

**Fig 4:**
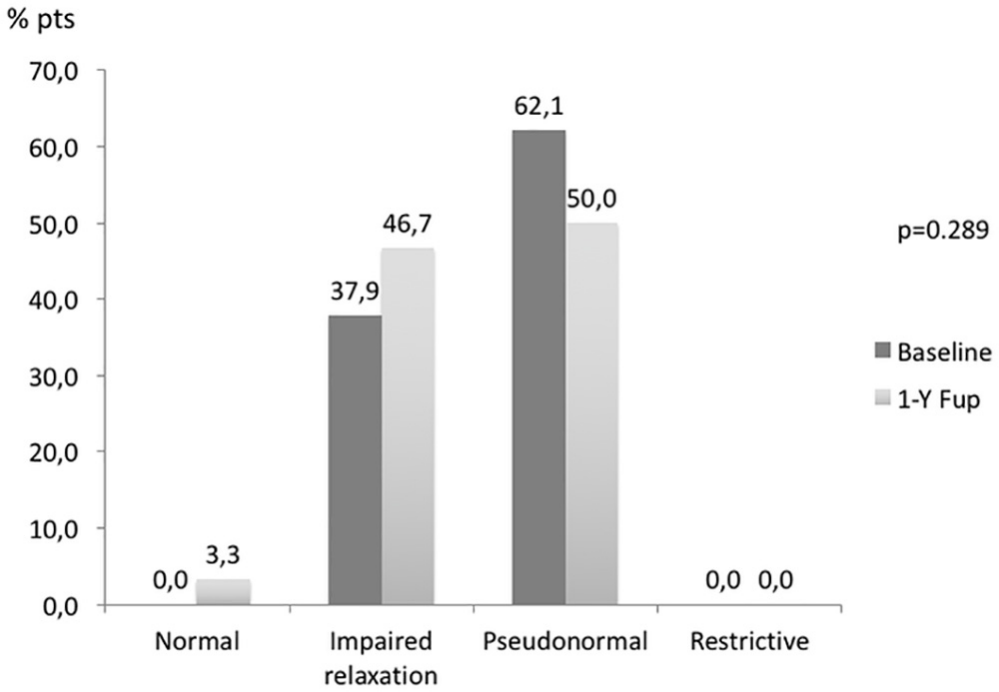
Comparison of LV diastolic function at baseline and 1 year after renal denervation. The percentage of patients in each diastolic function group (Normal, Impaired relaxation, pseudonormal and restrictive) is depicted.

LVDD was diagnosed in 29 (93.5%) patients, 11 (37.9%) of them had grade 1 diastolic dysfunction, 18 patients a pseudo-normal pattern (62.1%); 2 patients were in atrial fibrillation and there were no patients with a restrictive filling pattern (Fig 4). For the entire population, E/A ratio was 0.8±0.2, E-wave deceleration time 225±49ms and E/e’ ratio 11±3.

After one-year, there was an overall significant reduction in LV mass (152±32 to 136±34g/m^2^, p<0.001—Fig 2), an increase in mitral valve deceleration time (from 225±49ms to 247±51ms, p = 0.015—Table 4). There were no significant changes in the distribution of patients among the different LV geometric patterns (Fig 3) or in the percentage of patients in each diastolic function group (Fig 4) from baseline to 1 year after renal denervation.

**Table 4:** Echocardiographic parameters at baseline and at one-year follow-up in patients submitted to RDN.

	Baseline	One-year	p
**Anatomy**			
LVEDV (mL)	93.3±18,2	110.9±27.4	.004
LVESV (mL)	35.8±12.6	38.2±3.1	.121
IVSTd (mm)	13.4±1.9	13.1±2.4	.616
PWTd (mm)	11.7±1.6	11.8±1.7	.620
LVEDD (mm)	48.7±5.8	47.8±5.4	.230
LVESD (mm)	28.9±5.7	27.9±6.5	.296
LV mass/BSC (g/m^2^)	152.3±32.4	135.7±33.9	< .001
LA volume index (ml/m^2^)	32.8±8.3	34.1±6.2	.227
**Function**			
LVEF Simpson (%)	64.5±9.2	67.7±9.1	.001
Stroke volume (ml)	81.7±14.9	102.7±16.7	.075
Mitral valve E Vmax (cm/s)	73.6±15.2	73.2±16.4	.881
Mitral valve A Vmax (cm/s)	88.3±16.5	86.0±21	.469
Mitral valve E/A ratio	0.84±0.21	0.86±0.20	.574
Mitral valve E deceleration time (ms)	224.9±49.4	247.3±50.5	.015
Mitral valve lateral E’ (cm/s)	7.2±1.8	7.3±2.1	.417
Mitral valve lateral E/E’	11.0±3.3	10.5±3.5	.228

LVEDVI, left ventricle end-diastolic volume; LVESVI, left ventricle end-systolic volume; IVSTd, interventricular septum thickness on diastole; PWTd, posterior wall thickness on diastole; LVEDD, left ventricle end-diastolic diameter, LVESD, left ventricle end-systolic diameter; LV, left ventricle; BSC, body surface area; LA, left atrium; LVEF, left ventricle ejection fraction.

### Relation between blood pressure reduction and echocardiographic findings

**Fig 5:**
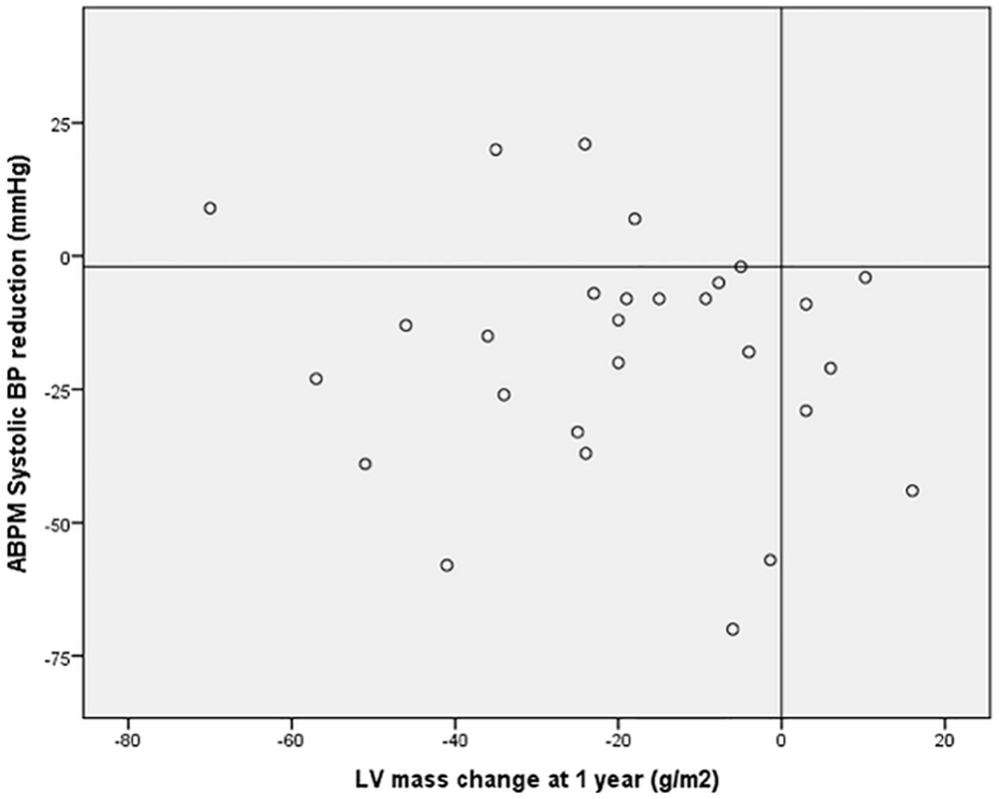
Relation between LV mass index and ABPM systolic BP changes at 1 year follow-up. Horizontal line set at 2mmHg for responder in ABPM systolic BP reduction. Five patients had regression in LVMI without significant (>2mmHg) reduction in ABPM systolic BP and 5 additional patients were ABPM systolic BP responders but without reduction in LVMI. BP- blood pressure; ABPM –24 hours ambulatory blood pressure measurement; LV—left ventricle.

Reduction in LV mass reached statistical significance in ABPM SBP responders (n = 26): 148±32 to 133±29g/m^2^, p<0.001. In non-responders (n = 5), LV mass also decreased: 166±23 to 129±15g/m^2^, p = 0.05, although not reaching statistical significance certainly due to sample size. From the scatter-plot graphic (Fig 5) where the relationship between LV mass and ABPM SBP changes at one year for the entire population is shown, we observe that changes in SBP and LV mass are not correlated, as depicted by the very low r2 values obtained.

### Safety

There were 3 mild hematomas and 1 femoral pseudoaneurysm, treated with surgery without any permanent sequelae. There were no complications related to the renal arteries, namely dissection or perforation.

## Discussion

The main findings of our study were: 1) Renal denervation in patients with resistant HTN was associated with significant reduction in both office and ABPM blood pressure at 1 year follow-up; 2) There was a significant reduction in left ventricle mass index, a recognized marker of HTN target organ damage.

Renal denervation has been associated with significant reductions in both office and ABPM blood pressure in many trials[2,3,17] and registries[18]. In a recent large randomized trial the reductions in systolic blood pressure, the primary endpoint of the trial, was not significant as compared to a sham control arm, in striking contrast with previous trial.[4] Many possible confounding factors were pointed out that could explain these apparent contradictory findings,[19] but most importantly these inconsistent results of renal denervation makes a strong case for additional studies looking beyond blood pressure measurements. With this rational we sought to evaluate the impact of renal denervation in left ventricle hypertrophy, which is one of the most important markers of target organ damage of HTN and has been associated with an increased rate of cardiovascular events and death independent of BP values[20–22]. At 1 year after renal denervation, there was a significant reduction in left ventricle mass and our results are in line with previous studies using both transthoracic echocardiogram [23,24] and cardiac magnetic resonance [25].

Brandt MC et al[24] in a study including 46 patients, found that renal denervation was associated with a significant reduction in LV mass index and filling and improvements in mitral valve lateral E/E´, indicator of LV filling pressure in transthoracic echocardiogram. In another small study using similar methodology, Schirmer SH et al [23] evaluated the impact of renal denervation in left ventricle hypertrophy by echocardiogram and were able to document that in patients with resistant HTN, the observed reductions in LV mass were similar across tertiles of systolic blood pressure, suggesting that the pathophysiology could be related also to a direct effect of sympathetic hyperactivity, not dependent on blood pressure or heart rate. In our registry we didn´t found a correlation between LV mass and ABPM SBP changes at one year (Fig 5), which suggests that LV hypertrophy reduction, after RDN, might be affected by other mechanisms beyond BP reduction. This is not new in the field of HTN and it has been previously described that for similar BP reduction, different pharmacological agents could lead to different impact on LV hypertrophy [26]. In one interesting study, a greater regression in LV hypertrophy was documented for a drug combination that targeted neuroendocrine activity (both renin-angiotensine-aldosterone system and sympathetic nervous system), for the same magnitude of BP reduction [27]. Regarding the pathophysiological mechanism of the observed reduction of LV mass, it could be the result not only of a reduction in myocyte hypertrophy but also of absolute collagen content and diffuse interstitial myocardial fibrosis, as was suggested in a recent cardiac MRI study [28].

In a multicenter study including 72 patients and using cardiac magnetic resonance imaging, Mahfoud F et al [25] also demonstrated that at 6 months follow-up renal denervation was associated with a significant reduction in left ventricle mass index, an improvement in ejection fraction and a reduction in left ventricle circumferential strain, a surrogate of diastolic function. Taken together these studies are consistent in regression of LV mass and improvement in several markers of diastolic function. In our study, we also found a significant reduction in LV mass but there were no significant changes in transthoracic echocardiogram parameters of diastolic function. In addition, we didn’t found any reduction in left atrial volume index. There was a small but significant increase in LV ejection fraction and LV end-diastolic volume, which could be explained at least partially by the numerically lower heart rate at 1 year follow-up documented both on office and on the average 24-hour heart rate from the ABPM recording. This small increase in EF is in line with some [24,25] but not all of the previous studies [23].

Some additional particular features of the present study should also be taken in consideration. First, our results come from a registry with a very rigorous selection process of patients for renal denervation, perceived from the high mean number (5.8) of antihypertensive drugs, the baseline office and ABPM blood values and the patient selection flowchart presented in Fig 1, with an almost 5:1 proportion of patients evaluated/treated (only 65 patients submitted to RDN out of the 318 with resistant HTN evaluated in our outpatient clinic). It is also worth mentioning that an average of 5.8 drugs is higher than that reported by other similar studies evaluating the impact of RDN on LV mass (ranging from 4.3 in the study of Schirmer SH et al [23] to 4.7 in the study of Brandt MC et al [24]. Secondly, in our study we have a very high percentage of patients taking spironolactone on baseline (74%). This high aldosterone antagonist use is in line with the described strict selection process, and in addition it might have also contributed to explain the positive results after renal denervation, since it has been demonstrated that patients previously treated with spironolactone where better responders to this procedure.[4,19] Thirdly, we used 24-hour ABPM in all patients and this is considered to be a more accurate evaluation of the impact of treatment on blood pressure.[10] Lastly, our results both in blood pressure and LV mass were evaluated at 1 year, a significantly longer follow up than that reported by the previous studies that evaluated patients at 6 months follow up.[23–25]

## Limitations

The present study has several limitations that should be acknowledged: 1) It is a single centre prospective registry with a small sample size. 2) The lack of a control group and the fact that there was no blinding either for RDN (sham not performed) or for the physicians performing the follow-up echocardiograms; 3) There were changes on antihypertensive drug therapy during the clinical follow-up which can influence the reductions in blood pressure and LV mass, although in our study the mean number of drugs was reduced. This way, the reduction obtained with renal denervation could have been underestimated in this real world setting; 4) No specific techniques were used to control for patient adherence to medication; 5) Echocardiograms were not reviewed in a core lab, which could potentially be associated with less reproducible measurements; 6) Cardiac MRI was not used and could have provided a more accurate evaluation of LV mass changes.

## Conclusions

In this single centre registry of patients with resistant hypertension, renal denervation was associated with significant reduction in both office and 24h-ABPM blood pressure, and a significant decrease in left ventricle mass evaluated by transthoracic echocardiogram at 1 year follow-up. There were no significant changes in left atrium volume index or in the distribution of patients among the different left ventricle geometric patterns and diastolic function subgroups.
